# Antimicrobial and Anti-Biofilm Activities of Citrus sinensis and Moringa oleifera Against the Pathogenic Pseudomonas aeruginosa and Staphylococcus aureus

**DOI:** 10.7759/cureus.12337

**Published:** 2020-12-28

**Authors:** Mohammad Zubair

**Affiliations:** 1 Medical Microbiology, University of Tabuk, Tabuk, SAU

**Keywords:** moringa oleifera, citrus sinensis extracts, s. aureus, pseudomonas species, tabuk city, esβl and mrsa production, biofilm formation

## Abstract

Context

The plant *Moringa oleifera* Lam (Moringaceae), generally termed as drumstick tree, and *Citrus sinensis* Linn (Rutaceae) fruit have the ability to treat multiple human infections. A biofilm is none other than a complicated microbial community whose nature is greatly resistant to antimicrobial elements. The development of biofilms in abiotic and biotic surfaces has a connection with higher levels of mortality and morbidity. Along with that, it is regarded as a vital element of bacterial pathogenicity.

Aim

The present study evaluated the inhibitory effect and anti-biofilm activity of *Moringa oleifera (M. oleifera) *and *Citrus sinensis (C. sinensis) *extracts against those of pathogenic *Pseudomonas aeruginosa (P. aeruginosa) *and* Staphylococcus aureus (S. aureus).*

Materials and methods

Two plant materials were collected from the local market of Tabuk city and two human pathogenic microbial strains were used in the study: *S. aureus* and *P. aeruginosa*. Further, a series of morphological, physiological, and conventional biochemical tests were performed to identify the selected microorganisms. In addition to this, the study conducted the following tests: antibiotic sensitivity test, extended-spectrum β-lactamase (ESβL), and methicillin-resistant Staphylococcus aureus (MRSA) production, biofilm formation in 96-well microtiter plates, minimum inhibitory concentration (MIC) determination, the effect of sub-MICs of *C. sinensis* extract and *M. oleifera* extract on the viability of test bacteria, and finally, measurement of the inhibition of biofilm.

Results

A remarkable result of the research is that the peel extract of *C. sinensis*and the flesh extract of *M. oleifera* efficiently inhibited biofilm formation by the addition of sub-inhibitory concentrations of (1/16 x MIC - 1/2 x MIC) MRSA and ESBL, respectively. *P. aeruginosa *shows high resistance to piperacillin (85.0%). Similarly, the resistance of MRSA was also high (65%) against gentamycin and amikacin antibiotics. Regarding ESBL, 12 (60%) isolates showed confirmed positive and 45% of *S. aureus* showed MRSA activity. On observing the 12 ESβL-positive *P. aeruginosa*, it was found that five strains (PS1, PS4, PS6, PS8, and PS11) have formed strong biofilm, methicillin-resistant *S. aureus* while four strains showed strong biofilm activity (SA2, SA4, SA5, and SA8). The MIC of *C. sinensis* extract and *M. oleifera* extract against strong biofilm producers had a range of 50-2000 µg/ml concentration after overnight incubation. The study results revealed that the antibiofilm activity comparatively showed the extract of *M. oleifera* was better than *C. sinensis*against the mixed culture (PS1+SA8, PS6+SA2, and PS8+SA4). Hence, it is recommended to use M. oleifera as an option to monitor the development of microbial biofilms or as a model for looking for better medicines.

Conclusion

The presence of antimicrobial activity found in *M. oleifera* and *C. sinensis* extracts offers convincing evidence of their likely action as antimicrobial metabolites against the studied microorganism. Anti-biofilm assay findings have shown that *M. oleifera* and *C. sinensis* extracts have effectively blocked MRSA and ESBL development in the biofilm matrix.

## Introduction

Antibiotics are being utilized for treating infections, in both animals and humans all over the world. Along with the therapeutic usage in animals, antibiotics are generally added in small quantities to animal feeds like prophylaxis and for the purpose of promoting growth [1]. A consistent increase has been noted in terms of microbial resistance to antimicrobials. In addition to that, a decreasing ability is found in the antimicrobials that are available for the purpose of treating general infections.

Antimicrobial resistance (AMR) is a vital risk when it comes to the health and welfare of the people as well as animals, which ends up with a great effect in terms of food security. Anyway, there is a dilemma all across the globe regarding the reduction of new therapeutic elements, which helps in treating different kinds of diseases that affect both animals as well as humans. There are chances for society to go through a post-antibiotic period with present antibiotics getting ineffective slowly because of resistance. This contains big threats in terms of health and national security as well; for instance, bioterrorism and pandemics [2]. The estimation taken at the present time discloses a death toll of about 700,000 people annually. This is mainly because of the antibiotic resistance and a forecast that by 2050, there are higher chances for about 10 million people to be at threat, provided no measures are taken in stopping the drift of rising AMR [3]. In addition to that, it's presumed that people who live in developing nations that are subjected to mortality and morbidity due to the impact of infectious diseases shall be the ones who would be deadly affected by this condition.

The formation of biofilm is one among the strategies of resistance by several pathogens that indeed makes them even more complicated as compared to the platonic counterparts [4]. A biofilm is none other than a complicated matrix of microorganism communities that consist of proteins, polysaccharides, and other organic elements, wherein cells get fixed together, forming powerful attachments to abiotic and biotic surfaces [5]. Biofilms allow microbes that bind to a surface in order to persist despite harsh states like antimicrobial agents and host defenses [6]. Thus, the formation of biofilm is one of the indirect sources of action through which bacteria act resistant in terms of antibiotics and this is where even resistance genes get transferred among biofilm micro-community members [7]. *P. aeruginosa* and *S. aureus* are two, vital, opportunistic-natured pathogens all over the world, which cause nosocomial and community-acquired infections. Biofilm-related infections are caused by *P. aeruginosa* and S. aureus, and methicillin-resistant *S. aureus* (MRSA) has evolved to be a pathogen (clinically appropriate) due to its nature of acting resistant toward antibiotics along with the potential of forming biofilms as examined by Alamri et al. [8]. Nearly 60% of microbial infections involve biofilms whilst 2/3rds of bacterial infections in humans are formed due to the biofilms [9].

*M. oleifera* Lam (Moringaceae) is a tree that has a fast-growing nature, and it is called drumstick tree or horseradish tree. A 4-(β-d-Glucopyranosyl-1→4-α-l-rhamnopyranosyloxy) - benzyl thiocarboxamide, which are separated from the seed has displayed the potential activity of antimicrobials as stated by Oluduro et al. [10]. In the traditional aspect, the report says that when equal parts of *C. sinensis* Linn (Rutaceae) fruit rind (orange peel) and *M. oleifera* roots are blended, it is beneficial for the bowels as stated by Gholap et al. [11]. Therefore, this work was planned to study the inhibitory effect and anti-biofilm activity of *M. oleifera* and *C. sinensis* extracts against the most common clinical isolates (*P. aeruginosa* and *S. aureus)*.

## Materials and methods

Plant samples 

Two plant materials were collected on the basis of traditional medicinal history from the local market of Tabuk city (Table 1).

**Table 1:** The botanical name, family, parts used, and ethnomedicinal use under this study

Botanical Name	Common name	Family name	Part used	Ethnomedicinal use
Citrus sinensis	Egyptian malta	Rutaceae	Peel	Treatment of cold, anorexia, and cough
Moringa oleifera	Horseradish tree, Radish tree, Drumstick tree	Moringaceae	Flesh	Curing of fever, infections in the ear, reduction of blood sugar and pressure

Collection of a bacterial sample

Forty bacterial samples (20 *S. aureus* and 20 *P. aeruginosa*) were used in the study. To be doubly sure, basic biochemical tests were performed. The antibiotic assay was performed using the Kirby Bauer disc diffusion method following the Clinical and Laboratory Standards Institute (CLSI) guideline.

Extraction of test samples (a) *C. sinensis* (Egyptian malta), (b) *M. oleifera*

The methodology of powder preparation of *C. sinensis* and *M. oleifera* was adopted from previously published reports [12]. The details are presented in the supplementary file.

Antibiotic sensitivity testing of *P. aeruginosa*. and *S. aureus*

Antibiotic sensitivity was performed on Mueller-Hinton agar by the Kirby-Bauer method. Amikacin (30 µg), ceftazidime(30 µg), cefepime (30 μg), levofloxacin (5 μg), tobramycin (10 µg), piperacillin (100 μg), “imipenem (10 μg), cefoperazone (75 μg), cefoperazone/sulbactam (75/10 μg), cefotaxime (30 µg), cefotaxime/clavulanic acid (30/10 µg), piperacillin/tazobactam (100/10 μg),” cefepime clavulanic acid (30/10 µg), sparfloxacin (5 μg), tobramycin (10 μg), “erythromycin (15 μg), gentamicin (10 μg), oxacillin (1 μg), ciprofloxacin (5 µg), cefoxitin (30 μg), and vancomycin (30 μg),” were used in this study (Hi-Media Labs, Mumbai, India]. Interpretation of results as suggested by the manufacturer's recommendation (Hi-Media Labs).

ESβL and MRSA detection among isolated strains

ESβL producers were detected by using ceftazidime and cefotaxime alone and in combination with clavulanic acid (10 mg), as recommended by the Clinical and Laboratory Standards Institute (CLSI) guidelines. For MRSA detection, the swab (sterile) was dipped in the *S. aureus *suspension (0.5 McFarland) and plated onto Mueller Hinton agar (MHA). Oxacillin discs (1 μg) were used with an overnight incubation at 30°C. When the zone of inhibition was ≤14 mm in diameter, it was considered resistant to oxacillin. The control strains used in this study were “*E. coli *ATCC 25922 (non-ESBL-producer), *K. pneumoniae* 700603 (ESBL-producer), *S. aureus* (ATCC 25923).”

Biofilm formation in 96-well microtiter plates

Biofilm formation was examined by the quantitative determination of biofilm formation in 96-well flat-bottom plates by Coffey and Anderson [13]. For each clinical strain tested, biofilm assays were performed in triplicate and the mean biofilm absorbance value was determined. The biofilms formed were classified as weak (OD590 0.1 to ≤0. 400), moderate (OD590 > 0.400), and strong (OD590 > 0.800).

Minimum inhibitory concentration (MIC) determination

The MIC of *C. sinensis* extract and *M. oleifera* extract against resistant biofilm-forming strains of *P. aeruginosa* and *S. aureus* was estimated using the standard micro-broth dilution method recommended CLSI guidelines at 37°C at 600 nm after 24 h incubation.

Effect of *C. sinensis* extract and *M. oleifera* extract on mono and mixed-species biofilms 

In inhibition assays, bacteria inoculated in microtitre plates were treated with 1/18-1/2 x MICs of *C. sinensis* extract and *M. oleifera* extract and incubated at 37 °C for 48 h. The inhibition of biofilm was measured as described in the previous section. The mixed biofilm formation was quantified as described by Zhang et al. [14].

Statistical analysis

Data represented in the manuscripts were mean value, experiments were performed in triplicate, and tests were analyzed using the student’s t-test.

## Results

Overall, 40 bacterial strains (20 *S. aureus* and 20 *P. aeruginosa*.) were collected from various hospitals in the city of Tabuk, Kingdom of Saudi Arabia. The collected bacteria were allowed to get subjected to the process of drug resistance observation (tabulated results are shown in Table 2).

**Table 2:** Antibiotic resistance pattern: a) P. aeruginosa ; b) S. aureus; c) ESBL pattern; d) MRSA pattern *P. aeruginosa*:* Pseudomonas aeruginosa*; *S. aureus*: *Staphylococcus aureus*; ESBL: extended-spectrum beta-lactamase; MRSA: methicillin-resistant Staphylococcus aureus

a) Resistance pattern	P. aeruginosa		b) Resistance pattern	S. aureus
	N=20			N=20
Amikacin	15(75.0)		Amikacin	13(65.0)
Ceftazidins	9(45.0)		Erythromycin	10(10.0)
Cefepime	14(70.0)		Ciprofloxacin	9(45.0)
Levofloxacin	15(75.0)		Gentamycin	13(65.0)
Sparfloxacin	15(75.0)		Levofloxacin	10(50.0)
Tobramycin	14(70.0)		Oxacillin	9(45.0)
Piperacillin	17(85.0)		Vancomycin	0(0)
c) ESBL pattern				
Preliminary test				
Ceftazidime		14 (70.0)		
Cefotaxime		15 (75.0)		
Confirmatory Test				
Ceftazidime/Ceftazidime Clavulanuc acid		12 (60.0)		
Cefotaxime/Cefotaxime Clavulanuc acid		12(60.0)		
d) MRSA results				
Oxacillin		9(45.0)		

Antibiotic resistance pattern

*P. aeruginosa* shows high resistance to piperacillin (85.0%) followed by amikacin, levofloxacin, and sparfloxacin, which show 75%, respectively. Similarly, the resistance of *S. aureus* was also high (65%) against gentamycin and amikacin antibiotics, respectively. Regarding ESBL, 12 (60%) isolates showed confirmed positive and 45% of S. aureus showed MRSA activity. For the antibiofilm activity of the *C. sinensis* extract and the *M. oleifera* extract, MRSA and ESβL-positive *P. aeruginosa* were selected for biofilm activity.

Biofilm activity of *P. aeruginosa* and *S. aureus*

**Figure 1:**
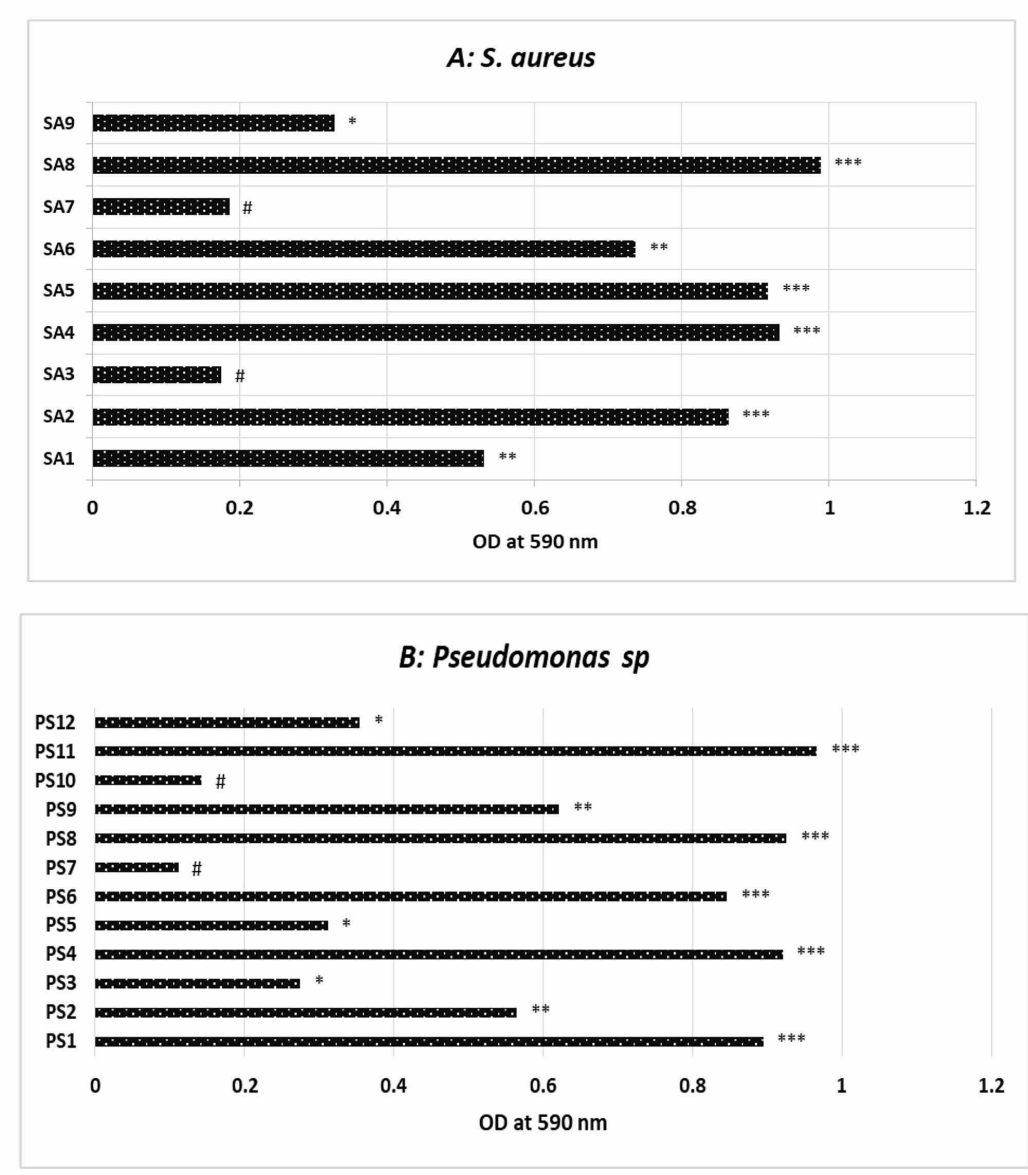
Mono-species biofilm formation among isolated strains [A] Methicillin-resistant *Staphylococcus aureus* (MRSA); [B] extended-spectrum beta-lactamase (ESβL)-producing *Pseudomonas sp.* ***, strong biofilm; **, Moderate biofilm; *, weak biofilm, # negative

The mono species biofilm formation among MRSA and ESβL-positive *P. aeruginosa* were classified as strong, moderate, weak, and negative (Table 3). On observing the 12 ESBL-positive *P. aeruginosa* strains, it is found that five strains (PS1, PS4, PS6, PS8, and PS11) have formed strong biofilms, two strains as moderate (PS2 and PS9), and two as weak biofilms (PS7 and PS10) (Figure 1A). In the case of methicillin-resistant *S. aureus*, four strains show strong biofilm activity (SA2, SA4, SA5, and SA8), whereas two strains (SA1, SA6) were moderate formers and SA9 was a weak biofilm producer (Figure 1B). For the antibiofilm activity of *C. sinensis* extract and *M. oleifera* extract, only the strong biofilm-positive methicillin-resistant *S. aureus* (SA2, SA4, SA5, and SA8) and *P. aeruginosa* (PS1, PS2, PS6, PS12, and PS18) were selected for further experiments.

**Table 3:** Classification of P. aeruginosa and S. aureus for biofilm activity as strong, moderate, and weak (data are n (%) unless otherwise indicated) *P. aeruginosa*:* Pseudomonas aeruginosa*;* S. aureus*:* Staphylococcus aureus*

	Biofilm producers			
	Strong n (%)	Moderate n (%)	Weak n (%)	Negative n (%)
P. aeruginosa (n=12)	5	2	3	2
S. aureus (n=9)	4	2	1	2

MICs of *C. sinensis* extract and *M. oleifera* extract

The MIC of *C. sinensis* extract and *M. oleifera* extract against strong biofilm producers with a range from 50-2000 µg/ml concentrations after overnight incubation are depicted in Table 4.

**Table 4:** MIC of Citrus sinensis extract and Moringa oleifera extract strong biofilm-forming ESBL-producing Pseudomonas and methicillin-resistant (MRSA) strains. MIC: minimum inhibitory concentration; ESBL: extended-spectrum beta-lactamase; MRSA: methicillin-resistant *Staphylococcus aureus*

Strains	MIC of Citrus sinensis extract (µg/ml)	Sub-MIC of Citrus sinensis extract (µg/ml) ^a^				MIC of Moringa oleifera extract (µg/ml)	Sub-MIC of Moringa oleifera extract (µg/ml) ^b^			
ESBL		1/16xMIC	1/8xMIC	1/4xMIC	1/2xMIC		1/16xMIC	1/8xMIC	1/4xMIC	1/2xMIC
PS1	200	12.5	25	50	100	1200	75	150	300	600
PS4	300	18.7	37.5	75	150	1000	62.5	125	250	500
PS6	100	6.25	12.5	25	50	800	50	100	200	400
PS8	400	25	50	100	200	1600	100	200	400	800
PS11	350	21.8	43.7	87.5	175	1000	62.5	125	250	500
MRSA										
SA2	100	6.25	12.5	25	50	800	50	100	200	400
SA4	400	25	50	100	200	1600	100	200	400	800
SA5	600	37.5	75	150	300	2000	125	250	500	1000
SA8	200	12.5	25	50	100	1200	75	150	300	600

**Figure 2:**
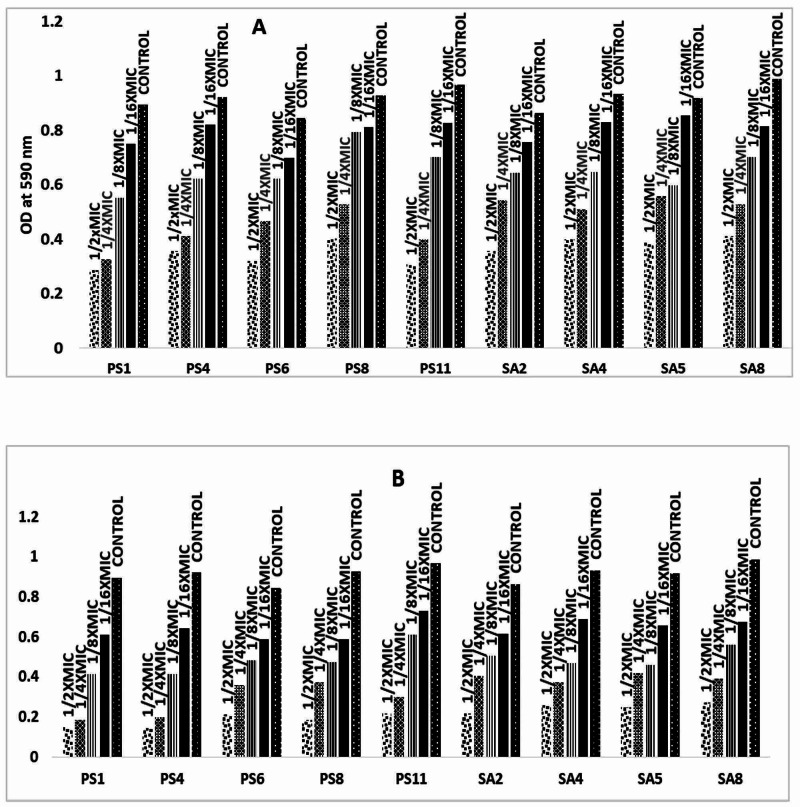
Inhibition of biofilm formation by sub-inhibitory concentrations of [A] C. sinensis extract; [B] M. oleifera extract. The data represent the mean values of three independent experiments. *C. sinensis*:* Citrus sinensis; M. oleifera*:* Moringa oleifera*

The sub-lethal concentrations dose were selected for biofilm inhibition assay (Table 4). The adding of extracts of *C. sinensis* and *M. oleifera* at respective 1/2 x MIC at the beginning of the growth showed no change in the growth of strong biofilm-positive MRSA (SA2, SA4, SA5, and SA8) (Figure 2A). A similar pattern was also observed for ESBL-positive, strong biofilm-producing *P. aeruginosa *(PS1, PS2, PS6, PS12, and PS18) for *C. sinensis* and *M. oleifera* at respective 1/2 x MIC (Figure 2B). To avoid the reduction of biofilm formation activity, this sub-MIC was performed.

Inhibition of mono-culture biofilm by *C. sinensis* extract and *M. oleifera* extract

Figure 2A bar graphs indicate the inhibition of biofilm formation by sub-inhibitory concentrations of *C. sinensis* extract (1/16 x MIC - 1/2 x MIC). The 14%-68% reduction of biofilm formation by ESBL-producing *P. aeruginosa* (PS11); 16%-68 % by PS1; 17%-62% by PS6; 61%-10% by PS4; and 12%-56% by PS8 as compared to control. The 12%-59% of MRSA (SA2); followed by SA8 (17%-58 %); SA4 (11%-57%), and SA5 (6%-57%) (Table 5, panel A).

**Table 5:** Reduction percentage of sub-inhibitory concentrations (for monoculture at 1/16xMIC-1/2xMIC; mixed culture at 1/16xMIC-1/8xMIC-1/4xMIC-1/2xMIC) of C. sinensis extract and M. oleifera extract against biofilm formation *C. sinensis*:* Citrus sinensis*;* M. oleifera*:* Moringa oleifera;* MIC: minimum inhibitory concentration

A: Monoculture								
Isolate Name	C. sinensis extract				M. oleifera extract			
	Reduction percentage at				Reduction percentage at			
	1/16xMIC		1/2xMIC		1/16xMIC		1/2xMIC	
PS1	16 %		68 %		31 %		86 %	
PS4	10 %		61 %		30 %		84 %	
PS6	17 %		62 %		30 %		75 %	
PS8	12 %		56 %		36 %		80 %	
PS11	14 %		68 %		24 %		77 %	
SA2	12 %		59 %		28 %		75 %	
SA4	11 %		57 %		25 %		72 %	
SA5	6 %		57 %		28 %		73 %	
SA8	17 %		58 %		31 %		72 %	
B: Mixed Culture								
Isolate Name	C. sinensis extract				M. oleifera extract			
	Reduction percentage at				Reduction percentage at			
	1/16xMIC	1/8xMIC	1/4xMIC	1/2xMIC	1/16xMIC	1/8xMIC	1/4xMIC	1/2xMIC
PS1+SA8	14 %	32 %	46 %	46 %	47 %	51 %	68 %	74 %
PS6+SA2	15 %	23 %	40 %	62 %	54 %	60 %	71 %	76 %
PS8+SA4	10 %	18 %	43 %	56 %	46 %	54 %	67 %	73 %

The bar graphs in Figure 2B graphs indicate the inhibition of biofilm formation by sub-inhibitory concentrations of *M. oleifera* extract (1/16 x MIC - 1/2 x MIC). The 31%-86% reduction of biofilm formation by ESBL-producing *P. aeruginosa* (PS1); 30%-84% by PS4; 36%-80% by PS8; 24%-77% by PS11; and 30%-75% by PS6 as compared to control. Then 28%-75% by MRSA (SA2); followed by SA5 (28%-73%); SA4 (25%-72%) and SA8 (31%-72%) (Table 5).

Inhibition of mixed biofilm

**Figure 3:**
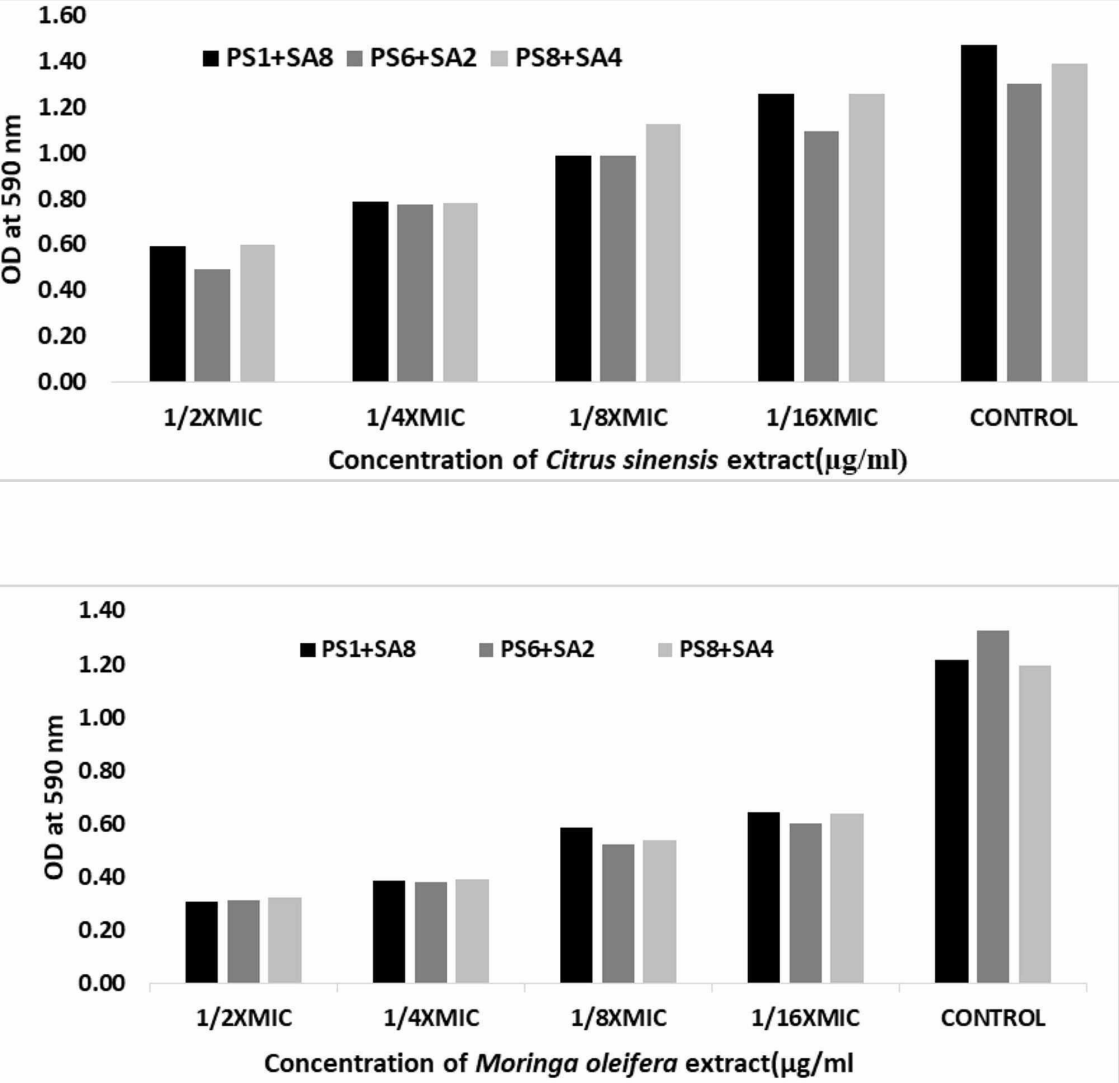
Effects of [A] C. sinensis extract, [B] M. oleifera extract on mixed biofilms. The data represent the mean values of three independent experiments *C. sinensis*:* Citrus sinensis*;* M. oleifera*:* Moringa oleifera*

Figure 3 (panels A-B) illustrates the comparative antibiofilm activity of *C. sinensis* extract and *M. oleifera *extract against the mixed culture (PS1+SA8, PS6+SA2, and PS8+SA4). The overall antibiofilm activity of *M. oleifera* was reported better as compared with *C. sinensis* (Table 5 panel B) when compared with controls at 1/16xMIC-1/8xMIC-1/4xMIC-1/2xMIC, respectively.

## Discussion

Biofilms are being identified as crucial in human disease, and the number of infections associated with biofilms is growing [6]. *S. aureus*, for example, has been shown to be among the most difficult pathogens implicated in a number of infections [15] such as indwelling medical device (IMD) infections associated with it. It has been noted that it is getting impossible to remove *Staphylococcus spp* biofilm infections when most of the drugs on the market have to be used as combination therapies [16]. Likewise, *P. aeruginosa* has arisen within immune-compromised individuals as one of the chief reasons for nosocomial infections [17].

The ESBL detection test showed 60% of *P. aeruginosa* were ESβL producers and the maximum of them showed high resistance to piperacillin (85.0%), which resembles the findings outlined by Harris et al. [18]. Piperacillin-tazobactam is commonly used in seriously ill patients to treat *P. aeruginosa* infections. The new U.S. medications are ceftazidime-avibactam and ceftolozane-tazobactam. Food and Drug Administration (FDA)-approved combinations of cephalosporin-β-lactamase inhibitors to treat Gram-negative bacilli-induced infections, including *P. aeruginosa* [19]. Similarly, the resistance of MRSA was also high (65%) against gentamycin and amikacin antibiotics, respectively, and 45% of *S. aureus* shows MRSA activity. The maximum isolates of *S. aureus* (84.5%) were comparable to other studies in terms of resistance to gentamycin [20].

The mono species biofilm formation among MRSA S. aureus and ESβL-positive *P. aeruginosa* were classified as strong, moderate, weak, and negative. On observing the 12 ESBL-positive *Pseudomonas strains*, it was found that five strains (PS1, PS4, PS6, PS8, and PS11) formed strong biofilms. In the case of methicillin-resistant *S. aureus*, four strains show strong biofilm activity (SA2, SA4, SA5, and SA8). Strong biofilm (referring to +++) showed a significantly higher likelihood of tolerance or resistance to antibiotics that will probably result in therapeutic failure in MRSA infections [21]. There is no prevailing opinion to date on the categorization of *S. aureus* isolates predicated on their biofilm-forming capability. The concept of a strong, medium, weak, and non-biofilm producer consequently varies widely between studies [22].

The MIC of *C. sinensis* extract and *M. oleifera* extract against strong biofilm producers is in the range of 50-2000 µg/ml concentrations after overnight incubation. Similar to our study, the study by Ahmad and Aqil [23] showed the MICs measured from 64 to 1024 μg/ml for cefotaxime, cefuroxime, ampicillin, and penicillin.

Adding the extracts of *C. sinensis* and *M. oleifera* at respective 1/2 x MIC at the beginning of the growth showed no change in the growth of strong biofilm-positive methicillin-resistant *S. aureus* (SA2, SA4, SA5, and SA8). A similar pattern was also observed for ESBL-positive, strong biofilm-producing *P. aeruginosa*. (PS1, PS2, PS6, PS12, and PS18) for *C. sinensis* and *M. oleifera* at respective 1/2 x MIC. The formation of biofilms depends on several variables, including the environment, nutrient availability, geographical origin, specimen forms, and properties of surface adhesion and genetic composition of the species [24]. The data may have been influenced by these variables and led to the high prevalence found in the current research. It is not, however, understood as to how these variables are concerned.

Biofilms include MRSA and ESBL with a defensive barrier to withstand antibiotic therapy. A remarkable result of the research is that the peel extract of *C. sinensis* and a flesh extract of *M. oleifera* efficiently inhibited the biofilm-formation by the addition of sub-inhibitory concentrations of (1/16 x MIC - 1/2 x MIC) MRSA and ESBL, respectively. This indicates the acquired effect was dose-dependent. The better biofilm reduction by ESBL-producing *P. aeruginosa* is observed at higher concentrations of *C. sinensis* extracts at 14%-68% in PS11. Similar findings have been observed by Abraham et al. [25], who documented that methanolic caper extraction substantially inhibited biofilm formation and extracellular polymeric substance (EPS) development in *Proteus mirabilis*, *P. aeruginosa*, *Serratia marcescens*, and *E. coli*. Likewise, the better biofilm reduction of MRSA is observed at higher concentrations at 12%-59% in SA2. A similar pattern was also observed in *M. oleifera* extracts (1/16 x MIC - 1/2 x MIC). The 31%-86% % reduction of biofilm formation by ESBL-producing *Pseudomonas* (PS1); 30%-84 % by PS4; 36%-80% by PS8; 24%-77% by PS11; and 30%-75% by PS6 as compared to controls. The 28%-75% by MRSA (SA2); followed by SA5 (28%-73 %), SA4 (25%-72%), and SA8 (31%-72%). The existence of previously identified flavonoids, such as quercetin, kaempferol, naringenin, and apigenin, which are capable of reducing biofilm synthesis because they can suppress the activity of the autoinducer-2 responsible for cell-to-cell contact, can explain the inhibition of biofilm formation [26]. The inhibitory activity demonstrated by *C. sinensis* and *M. oleifera* could be derived from its ability to synthesize metabolites that can prevent the formation of biofilms. This result, however, needs to be further investigated through sophisticated qualitative and quantitative studies. A literature survey reveals that the antibiofilm activity by sub-inhibitory concentrations of *C. sinensis* and *M. oleifera* against MRSA and ESBL is scanty. Though, the antibiofilm perspective of flavonoids extracted from *M. oleifera* seed coat in contradiction to *S. aureus* is stated from India [27].

Our findings concerning the function of antibiofilms agree with previous work carried out in other terrestrial plant species from various parts of the world. Research from India, for example, reported that the *Vetiveria zizanioides* root extract showed an inhibition reduction in MRSA biofilm formation [28]. Similarly, another study conducted in Brazil found that *Piper regnellii*'s dichloromethane extract weakens biofilm formation [29].

The mono and mixed-species biofilm activity was quantified against *M. oleifera* and *C. sinensis*. The findings of the antibiofilm activity comparatively showed that the extract of *M. oleifera *was reported better as compared with *C. sinensis* against the mixed culture (PS1+SA8, PS6+SA2, and PS8+SA4). Hence, it is recommended to use *M. oleifera* as an option to monitor the development of microbial biofilms or as a model for looking for better medicines. Clinically, biofilm infections are significant, whereas bacteria show recalcitrance to antimicrobial compounds. Large concentrations of antimicrobials may be required to eliminate biofilm producers. Owing to the possibility of toxicity and the associated side effects, this might not always be feasible in vivo, however, low-concentration combination therapies can, therefore, be effective in eradicating *staphylococcal* biofilm-related infections, including those induced by MRSA [30]. For the selection of a suitable antimicrobial agent, the early screening and detection of biofilm producers followed by their antimicrobial susceptibility tests is essential.

## Conclusions

In the present study, according to my knowledge, this was the first report of the antimicrobial and antibiofilm activity of *M. oleifera* and *C. sinensis* in the Kingdom of Saudi Arabia. This study also provides better efficacy of *M. oleifera* over *C. sinensis* in its antibiofilm activity in both mono and mixed culture in vitro experiments. Further studies are required on the molecular mechanism involved in controlling the resistance by *M. oleifera. *It could, therefore, be concluded that new groups of anti-biotic leads will be provided by the bioassay-guided fractionation and purification of *M. oleifera* and *C. sinensis*.

## Appendices

Preparation of *C. sinensis* peel powder

*C. sinensis* (oranges) was procured from the local fruit market and any extraneous materials were first by washing. The orange peel was resized into 1x1 inch and dried at 40°C till its moisture content reduced to <5%. It was finely ground into powder. The 60 gram of fine powder of peel powder was dissolved in 160 ml of absolute ethanol at room temperature for three successive days. Using Whatman paper, the supernatant was filtered, and residues were used for a second and third extraction. For three days, dissolved parts were filtered and stored in a glass bottle. After the third extraction, the filtrates were then evaporated under reduced pressure at 50^∘^ C using a rotary evaporator to yield the crude extract [12].

Percentage Yield (%) = [Dry weight of the extract / Dry weight of plant material] × 100

The crude extract was collected in a vial for further use.

Preparation of *M. oleifera* (fruit flesh) powder

*M.  oleifera* was procured from the local fruit market and any materials were first by washing. The flesh was resized into 1x1 inch and dried at 40°C till its moisture content reduced to <5%. It was finely grounded into a powder. The 60 gram of fine powder of peel powder was dissolved in 160 ml of absolute ethanol at room temperature for three successive days. Using Whatman paper, the supernatant was filtered and residues were used for a second and third extraction. For three days, dissolved parts were filtered and stored in a glass bottle. After the third extraction, the filtrates were then evaporated under reduced pressure at 50^∘^ C using a rotary evaporator to yield the crude extract [12].

Percentage Yield (%) = [Dry weight of the extract / Dry weight of plant material] × 100

The crude extract was collected in a vial for further use.

Effect of *C. sinensis* extract and *M. oleifera* extract on mono and mixed-species biofilm

In brief, each strain (*S. aureus *and *P. aeruginosa*) was grown overnight at 37° C in tryptic soy broth (TSB) and diluted to 1 x 106 CFU/ml in the TSB. Equal numbers (1:1) of each bacterium were mixed together and the mixed bacterial suspension (100 µl) was added to each well of the polystyrene 96-well tissue-culture plates. Each well was filled with 100 µl of fresh TSB containing different concentrations of *C. sinensis* extract (1/16-1/2 x MIC). Negative control wells contained TSB only, and wells with no additives were used as positive controls. After incubation for 48 h at 37° C, plates were gently washed with 1X phosphate-buffered saline (PBS; pH 7.4) and stained with 100 μl of 0.1% crystal violet (CV) (Sigma-Aldrich, St. Louis, MO) for 30 min at room temperature. Excess CV was removed by washing, and biofilm was quantified by measuring the corresponding OD590 nm of the supernatant following the solubilization of CV in 95% ethanol. The experiment was repeated with *M. oleifera* extract effect on mono and mixed biofilm study [14].

## References

[1] Hashemi SR, Davoodi H (2011). Herbal plants and their derivatives as growth and health promoters in animal nutrition. Vet Res Commun.

[2] Lowrence RC, Ramakrishnan A, Sundaramoorthy NS (2018). Norfloxacin salts of carboxylic acids curtail planktonic and biofilm mode of growth in ESKAPE pathogens. J Appl Microbiol.

[3] Yelin I, Kishony R (2018). SnapShot: antibiotic resistance. Cell.

[4] de la Fuente-Núñez C, Korolik V, Bainset M (2012). Inhibition of bacterial biofilm formation and swarming motility by a small synthetic cationic peptide. Antimicrob Agents Chemother.

[5] Bazargani MM, Rohloff J (2016). Antibiofilm activity of essential oils and plant extracts against Staphylococcus aureus and Escherichia coli biofilms. Food Control.

[6] Jamal M, Ahmad W, Andleeb S (2018). Bacterial biofilm and associated infections. J Chinese Med Assoc.

[7] Lebeaux D, Ghigo JM, Beloin C (2014). Biofilm-related infections: bridging the gap between clinical management and fundamental aspects of recalcitrance toward antibiotics. Microbiol Mol Biol Rev.

[8] Alamri AM, Alsultan AA, Ansari MA, Alnimr AM (2015). Biofilm-formation in clonally unrelated multidrug-resistant Acinetobacter baumannii isolates. Pathogens.

[9] de la Fuente-Núñez C, Cardoso HM, de Souza Cândido E (2016). Synthetic antibiofilm peptides. Biochim Biophys Acta Biomembr.

[10] Oluduro OA, Aderiye BI, Connolly JD, Akintayo ET, Famurewa O (2010). Characterization and antimicrobial activity of 4-(β-d-glucopyranosyl-1→4-α-l-rhamnopyranosyloxy)-benzyl thiocarboxamide; a novel bioactive compound from Moringa oleifera seed extract. Folia Microbiol.

[11] Gholap PA, Nirmal SA, Pattan SR, Pal SC, Mandal SC (2012). Potential of Moringa oleifera root and Citrus sinensis fruit rind extracts in the treatment of ulcerative colitis in mice. Pharm Biol.

[12] Masad AA, Bashiti TA, Mosleh F, Abu Madi UM (2019). Antibacterial and antibiofilm activity of selected plant extract against some human pathogenic microorganism. Pak J Nutr.

[13] Coffey BM, Anderson GG (2014). Biofilm Formation in the 96-Well Microtiter Plate. Pseudomonas Methods and Protocols.

[14] Zhang QQ, Ye KP, Wang HH, Xiao HM, Xu XL, Zhou GH (2014). Inhibition of biofilm formation of Pseudomonas aeruginosa by an acylated homoserine lactones-containing culture extract. LWT - Food Sci Technol.

[15] Taylor TA, Unakal CG (2020). Staphylococcus aureus. StatPearls Publishing.

[16] Khatoon Z, McTiernan CD, Suuronen EJ, Mah TF, Alarcon EI (2018). Bacterial biofilm formation on implantable devices and approaches to its treatment and prevention. Heliyon.

[17] Pachori p, Gothalwal R, Gandhi P (2019). Emergence of antibiotic resistance Pseudomonas aeruginosa in intensive care unit; a critical review. Genes Dis.

[18] Harris AD, Perencevich E, Roghmann MC, Morris G, Kaye KS, Johnson JA (2002). Risk factors for piperacillin-tazobactam-resistant Pseudomonas aeruginosa among hospitalized patients. Antimicrob Agents Chemother.

[19] Allergan Allergan (2016). Allergan, Avycaz (ceftazidime-avibactam) for injection for intravenous use. California, United States, 2016. https://www.accessdata.fda.gov/drugsatfda_docs/label/2017/206494s003lbl.pdf.

[20] Khosravi AD, Jenabi A, Montazeri EA (2017). Distribution of genes encoding resistance to aminoglycoside modifying enzymes in methicillin-resistant Staphylococcus aureus (MRSA) strains. Kaohsiung J Med Sci.

[21] 21] Manandhar S, Singh A, Varma A, Pandey S, Shrivastava N (2018). Biofilm producing clinical Staphylococcus aureus isolates augmented prevalence of antibiotic resistant cases in tertiary care hospitals of Nepal. Front Microbiol.

[22] Crémet L, Corved S, Batard S (2013). Comparison of three methods to study biofilm formation by clinical strains of Escherichia coli. Diagn Microbiol Infect Dis.

[23] Ahmad I, Aqil F (2007). In vitro efficacy of bioactive extracts of 15 medicinal plants against ESβL-producing multidrug-resistant enteric bacteria. Microbiol Res.

[24] Kokare CR, Chakraborty S, Khopade SN, Mahadik KR (2009). Biofilm: importance and applications. Indian J Biotechnol.

[25] Sánchez E, Rivas Morales C, Castillo S, Leos-Rivas C, García-Becerra L, Ortiz Martínez DM (2016). Antibacterial and antibiofilm activity of methanolic plant extracts against nosocomial microorganisms: evidence-based complement. Altern Med.

[26] Vikram A, Jayaprakasha GK, Jesudhasan PR, Pillai SD, Patil BS (2010). Suppression of bacterial cell-cell signalling, biofilm formation and type III secretion system by citrus flavonoids. J Appl Microbiol.

[27] Onsare JG, Arora DS (2015). Antibiofilm potential of flavonoids extracted from Moringa oleifera seed coat against Staphylococcus aureus, Pseudomonas aeruginosa and Candida albicans. J Appl Microbiol.

[28] Kannappan A, Gowrishankar S, Srinivasan R, Pandian SK, Ravi AV (2017). Antibiofilm activity of Vetiveria zizanioides root extract against methicillin-resistant Staphylococcus aureus. Microb Pathog.

[29] Brambilla LZS, Endo EH, Cortez DAG, Dias Filho BP (2017). Anti-biofilm activity against Staphylococcus aureus MRSA and MSSA of neolignans and extract of Piper regnellii. Rev Bras Farmacogn.

[30] Wu WS, Chen CC, Chuang YC (2013). Efficacy of combination oral antimicrobial agents against biofilm-embedded methicillin-resistant Staphylococcus aureus. J Microbiol Immunol Infect.

